# Inclusion of persons with disability in sport: part 2 – best practices and policy recommendations from Qatar

**DOI:** 10.1136/bjsports-2022-106225

**Published:** 2022-09-16

**Authors:** Josélia Neves, Sanaa Taha Al-Harahsheh, Kamilla Swart, Sabika Shaban, Ali Hudaib

**Affiliations:** 1 College of Humanities and Social Sciences, Hamad Bin Khalifa University, Doha, Qatar; 2 World Innovation Summit for Health, Qatar Foundation, Doha, Qatar; 3 College of Science and Engineering, Hamad Bin Khalifa University, Doha, Qatar; 4 College of Islamic Studies, Hamad Bin Khalifa University, Doha, Qatar; 5 Al Shafallah Center for Persons with Disability, Qatar Foundation for Social Work, Doha, Qatar

**Keywords:** Sport, Recreation, Physical activity, Education, Public health

The upcoming FIFA World Cup 2022 has been announced as the most accessible for persons with disabilities (PWDs) in the history of international FIFA tournaments,^1^ a fact that will have a positive impact on the built environment and the lives of local communities. Such a major sporting event may galvanise the population to undertake greater physical activity, a contribution against obesity and sedentarism in the country. The commitment to a healthier population led to the establishment of National Sports Day, the only public holiday beyond Qatar National Day.

It is now acknowledged that all persons have the need and the right to engage in sport, and that PWDs are entitled to adaptations to access sport on equitable terms. Regrettably, there is no centralised data on the participation of PWDs in sport at the national level. The data gathered by different organisations makes comparisons and generalisations impossible. The purpose of this editorial is to offer a brief overview of PWDs’ engagement with sports in Qatar, including CWD, participation in elite parasport, and policy and infrastructure engagement.

## Physical therapy and education in Qatar

In the case of CWDs, early intervention will be the gateway to physical activity, and physical therapy should be individualised to develop the child at multiple levels. For instance, psychomotor therapy should combine emotional, physical and cognitive development; and play therapy may meet sensory, physical or behavioural challenges. The services listed in the Qatar Educational Directory^2^ show how physical therapies are integral to special education offered at high-quality state and independent schools, among which are the Al-Shafallah Center, Renad Academy and Al Noor Institute. CWDs attending inclusive (mainstream) education are offered adapted physical education.

Despite efforts to provide physical education to CWDs, there are few options for recreational activity. PWDs are less likely to travel, play non-competitive games, or simply share outdoor spaces,^3^ which afford physical activity that would enhance physical, emotional and social well-being.^4^ In 2017, Qatar gained its first accessible playground in Al Legtaifiya Family Public Park. Qatar’s first accessible swimming programme was established in 2012, and later hosted by Aspire Academy in 2016. The American School of Doha organises a Challenger Division within its Little League divisions to encourage CWDs to engage in baseball, with the help of student ‘buddies’. The Deaf Cricket Sports Center in Doha trains players to compete internationally, while the Qatar Foundation has the Ability Friendly Sports Program^5^ offering unique sport experiences. However, these opportunities remain inaccessible to the population for reasons such as cost, logistics, language, gender, disability and/or citizenship.

Box 1 **Qatar Foundation Ability Friendly Sports Program (AFP)**

The Qatar Foundation AFP is an initiative that was launched officially by Qatar Foundation in 2019 to support people with autism, learning disabilities, hearing disabilities, visual impairments and physical disabilities to take part in sports and developmental activities. The programme offers football and swimming classes, as well as seasonal camps that are tailored to each participant’s needs. The programme is open to participants aged three and older. The idea for the programme initially inspired by the World Innovation Summit for Health 2016 report ‘Autism: A Global Framework for Action’. This report contributed to Qatar Foundation’s own policies surrounding inclusion and ability friendly programmes, which ultimately led to the implementation of such programmes across Qatar.

Since delivering its first class in 2018, the programme has played an undeniable role in improving the quality of life for children on the autism spectrum by filling in a critical need for sports-based programmes. Aside from capturing the attention of families with children with autism, the sports programme also attracted strong interest from families with children of other abilities. The present-day AFP incorporates swimming and football lessons that are adapted to children of all abilities and ages. 1175 PWDs have participated in AFP sport activities between 2019 and 2022 (731 in swimming and 444 in football). About 75% of the participants are male (880), with ages ranging from 3 to 36. The majority of participants have autism (863). Additionally, there are 140 students with learning disabilities, 120 with physical disabilities, 49 with Down syndrome, 14 with hearing impairments, and 8 with vision impairments. Since its inception, the AF has delivered 16 550 sessions.^8^

To further support the programme’s growth and dissemination across and beyond Qatar, in collaboration with the College of Humanities and Social Sciences, Hamad bin Khalifa University, the AF programme has launched a Coaching the Coach Training Program,^9^ providing ‘theoretical and practical participant-centred learning, to develop the required competencies for coaching children with disabilities.’

### Competitive (elite/professional) parasport

Competitive parasport follows the Olympic motto ‘faster, higher, stronger’. In PWDs, comparability and classification derive from the athletes’ profiles, the modality and environmental adaptations. Parasport includes adapted versions of mainstream sport for non-disabled practitioners and modalities specifically for PWDs. The Qatar Olympic Committee works with national sports federations and the Qatar Paralympic Committee to promote inclusivity through sport, encouraging participation in national and international events. The National Federation for Sport with Disabilities has developed sport-specific frameworks providing adequate infrastructure and support, such as trained and qualified coaches, rehabilitative services, sports medicine, transport, equipment accessibility and communications.^6^

Box 2 **Qatar’s achievements in Paralympic Games**

Qatar made its debut at the 1996 Paralympic Games and has participated in every edition since then, with the first women participating in 2016. Twelve Qatari Paralympians also competed in the Asian Para Games 2014 in Incheon, South Korea, where Qatar won five medals (three gold, two bronze). As a measure of its strong commitment for sports for persons with disabilities, Qatar has hosted the October 2015 International Paralympic Committee (IPC) Athletics World Championship.^6^ Qatar won its first medals in the 2016 Games (men’s and women’s shot put) as well as bronze medal at the 2020+1 Games (men’s shot put). At the London 2017 IPC Athletics World Championships, team Qatar won its first gold medal and the first medal (silver) for women at the World Championships.

### FIFA Arab Cup 2021 and FIFA World Cup 2022 contributions to social change

**Figure 1:**
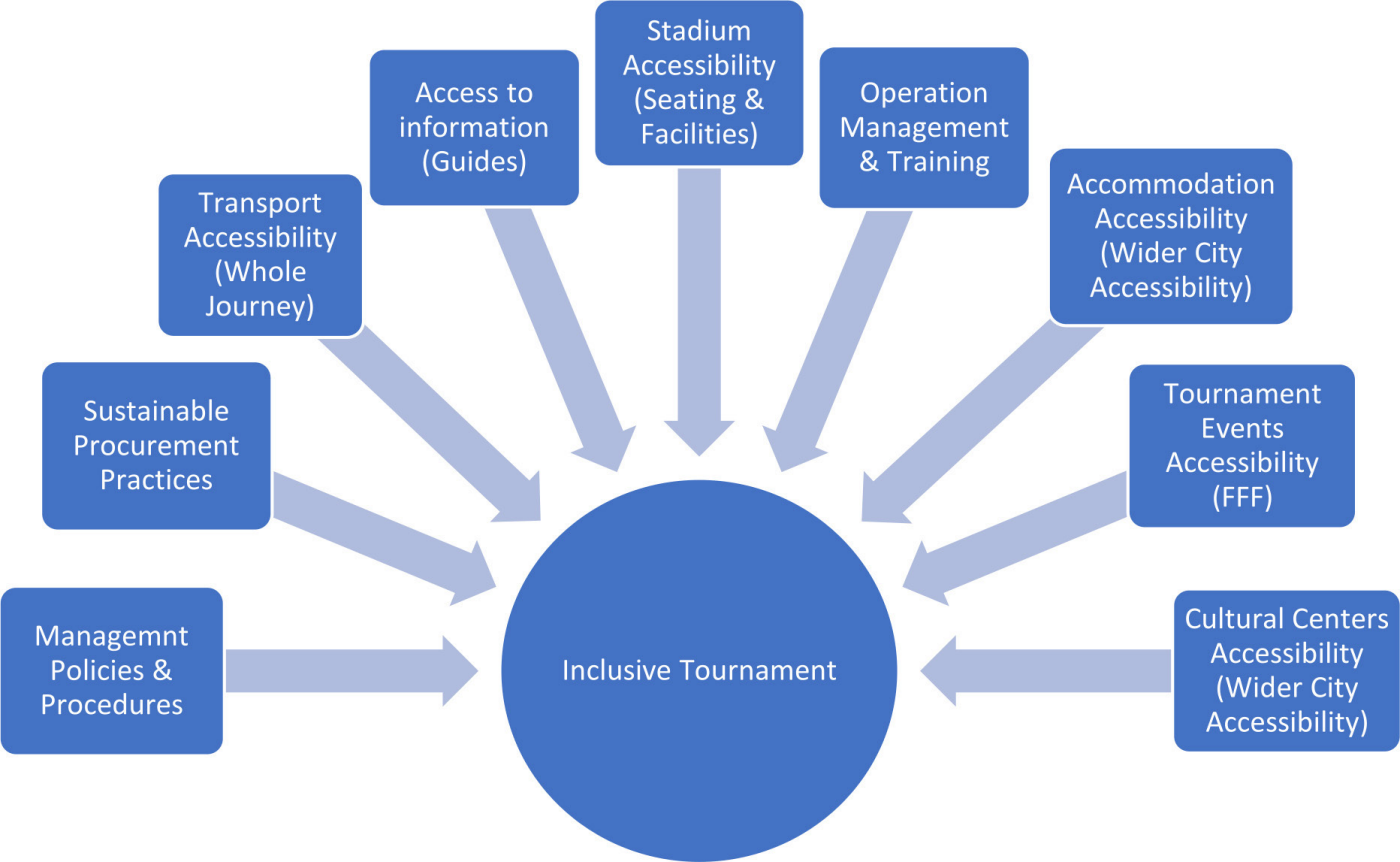
Inclusivity and accessibility areas. Source: Supreme Committee for Delivery and Legacy.

Sport brings people together to compete and collaborate. The mega sport events Qatar is hosting has leveraged significant social change in the country. The Supreme Committee for Delivery and Legacy (SC) has been entrusted with ‘overseeing the planning and development of host country operations’.^7^ In its vision, the SC commits to inclusivity by operationalising multiple areas (figure 1).

In 2016, the SC established the accessibility forum (AF), bringing together PWDs, experts and stakeholders to advise the SC and its stakeholders, review plans, provide experiential advice, and issue recommendations to relevant technical teams and partners. In 2018, a delegation of 15 AF members travelled to Moscow to assess the fan experience from arrival to departure to make recommendations for the Qatar tournament.

In December 2021, the FIFA Arab Cup tested the country’s preparedness to welcome fans of all abilities. Basic accessibility infrastructures and services are now guaranteed, and the country is ready for the FIFA World Cup 2022. All stadiums and precincts have been checked for access; several stadiums (Education City, Khalifa International and Al Janoub) have sensory viewing rooms for fans with sensory requirements; audio descriptive commentaries in Arabic and English are offered to blind fans; and volunteers and staff, some with disabilities themselves, have attended specific training to support fans with specific needs.

### Final thoughts and recommendations

The purpose of sport inclusion is to improve the quality of life of PWDs, and to stimulate social interaction. To support the health, well-being and inclusion of PWDs through sport, Qatar should address policies; financial investment and support; strategic planning; research, data collection, monitoring, and evaluation systems; qualification and training of providers; improvement of infrastructures; development of antistigma and awareness programmes; and the involvement of PWDs in the creation of sports initiatives.

Only multidisciplinary, transversal and collaborative effort will lead to the leveraging of a truly healthy and inclusive society, whose well-being is anchored in physical activity and sport.
